# Elevated level of neuroserpin is an indication for the resistance to gambogic acid-induced apoptosis and oxidative stress in triple-negative breast cancer cells

**DOI:** 10.2478/abm-2024-0010

**Published:** 2024-04-30

**Authors:** Ertan Kucuksayan, Hakan Kucuksayan, Mehmet Enes Sozen, Aslinur Sircan-Kucuksayan

**Affiliations:** Department of Medical Biochemistry, School of Medicine, Alanya Alaaddin Keykubat University, Alanya 07425, Turkey; Department of Medical Biology, School of Medicine, Kastamonu University, Kastamonu 37200, Turkey; Department of Histology and Embryology, School of Medicine, Alanya Alaaddin Keykubat University, Alanya 07425, Turkey; Department of Biophysics, School of Medicine, Alanya Alaaddin Keykubat University, Alanya 07425, Turkey

**Keywords:** apoptosis, gambogic acid, neuroserpin, oxidative stress, triple-negative breast cancer

## Abstract

**Background:**

The triple-negative breast cancer (TNBC) subtype, characterized by loss of HER2, estrogen, and progesterone receptors, displays aggressive phenotype and poor prognosis compared to other BC subtypes. Since the TNBC cells are devoid of receptors, endocrine therapy is an ineffective option for TNBC patients, necessitating canonical chemotherapy strategies to treat TNBC. It is crucial to use alternative and natural agents to support chemotherapy in TNBC.

**Objectives:**

To clarify the molecular mechanism of the tumorigenic effects of gambogic acid (GA) on TNBC cells with different epithelial character since GA has a wide spectrum of anticancer activity for most cancer types.

**Methods:**

We determined the cytotoxic dose of GA incubation of TNBC cells (MDA-MB-231 and BT-20 cells) for 24 h. We performed the MTT test and toluidine blue (TB) staining protocol for TNBC cells. We analyzed E-cadherin, N-cadherin, Bax, and neuroserpin mRNAs in both cells by qPCR. We evaluated apoptosis using DAPI staining and assessed the ROS using the 2ʹ,7ʹ-dichlorofluorescin diacetate (DCFH-DA) method.

**Results:**

We determined the IC_50_ concentrations of GA in MDA-MB-231 and BT-20 cells to be 315.8 nM and 441.8 nM, respectively. TB staining showed that BT-20 cells survive at excessive cytotoxic doses of GA, while most of the MDA-MB-231 cells were killed. Also, we found that BT-20 cells are more resistant to GA-induced apoptosis and oxidative stress than the MDA-MB-231 cells. qPCR results showed that GA upregulated neuroserpin, an oxidative stress-relieving factor in the BT-20 cells, but not in the MDA-MB-231 cells.

**Conclusions:**

The elevated level of neuroserpin could be a predictive marker to determine the development of resistance to chemotherapeutic agents.

Breast cancer (BC) is the most-commonly diagnosed cancer and the second common cause of cancer death in women. BC incidence has been increasing and is predicted to continue increasing [1]. The World Health Organization (WHO) estimates that there will be 805,116 female deaths in the world by 2030 and that BC will be the most-common type of cancer [2]. Chemotherapy and endocrine therapy are the still known most important and effective treatment approaches for BC patients. However, BC patients often develop resistance to chemotherapeutic drugs, leading to relapse of the cancer [3]. One of the causes of severe malignancy resistance to treatment is the development of resistance to apoptosis induced by chemotherapeutic drugs [4]. Carcinogenesis of BC depends on many factors such as the levels of the receptors of hormones, the loss of function of tumor suppressor genes, and the activation of oncogenes [5]. There are many types of BCs, and while most of these subtypes are curable, triple-negative breast cancer (TNBC) is the most aggressive and resistant to treatment, representing approximately 10%–15% of all BC cases [6, 7]. TNBC does not have estrogen or progesterone receptors and cannot express human epidermal growth factor receptor 2 (HER2). The tests for three proteins are negative in TNBC and differ from other types of invasive BC. Therefore, TNBC is more aggressive, its treatment options are limited, and has a worse prognosis [8].

Current chemotherapeutics inhibit tumor growth by killing cancer cells, but many problems are encountered in the clinic due to the side effects caused by the toxicity of these agents in healthy cells, leading to limiting the cancer-treatment options. In recent years, research on natural compounds has been increasing due to the development of drug resistance and the necessity to find strategies to desensitize cancer cells to chemotherapeutics [9, 10].

Natural compounds can kill cancer cells and have significantly less cytotoxic effects on healthy cells compared to chemotherapeutic drugs [11, 12]. Therefore, natural compounds could contribute to the development of different strategies to treat cancer since they may be less harmful than chemotherapeutics. Gambogic acid (GA) is xanthonoid derived from *Garcinia hanburyi*, also known as red mango, mainly found in Cambodia, Thailand, and southern Vietnam. GA has antioxidant and anti-inflammatory effects [13, 14]. GA can raise highly reactive oxygen species (ROS), impair cellular redox homeostasis, and lead to apoptotic and ferroptotic death in prostate cancer [15]. Moreover, GA has anti-invasive, antiangiogenic, and apoptotic effects on cervical and ovarian cancers [16, 17]. It has been shown that there may be an important anticipation for its use in treatment as an anticancer agent since GA has a capability to be implicated in many oncogenic signaling pathways of cancer cells. GA has also apoptotic effects because it can increase apoptotic factors like wild-type p53 and suppress antiapoptotic factors like B-cell lymphoma 2 (Bcl-2). It has anti-angiogenic effects by inhibiting platelet-derived growth factor (PDGF), epidermal growth factor (EGF), and vascular endothelial growth factor (VEGF). GA has also anti-invasive effects provided by decreasing the expression of matrix metalloproteinases (MMP) 2 and 9 via the protein kinase C (PKC) pathway [18]. However, the genotoxic and cytotoxic effects of GA on TNBC are unclear. Thus, the aim of our study was to clarify the molecular mechanism of GA-induced apoptosis in TNBC. Our results show that BT-20 cells are more resistant to GA compared to the MDA-MB-231 cells. Additionally, neuroserpin, an alleviating molecule for oxidative stress, was upregulated in GA-resistant BT-20 cells. This upregulation contributes to the survival of TNBC cells by reducing oxidative stress during GA treatment.

## Materials and methods

### Cell culture and treatment

MDA-MB-231 and BT-20 cells were cultured as TNBC cell lines. Both cells were purchased from American Type Culture Collection (ATCC). MDA-MB-231 and BT-20 cells were grown in Roswell Park Memorial Institute (RPMI) 1640 medium and in Eagle’s minimal essential medium (EMEM), respectively, supplemented with fetal bovine serum (FBS) (Sigma, Germany), 1% l-glutamine (Sigma, Germany), and 1% penicillin/streptomycin (Sigma, Germany). Cells were incubated with 5% carbon dioxide and at 37 °C in a humidified atmosphere. GA was purchased from Sigma Aldrich Chemicals and dissolved in 100% dimethyl sulfoxide (DMSO). The final concentration of DMSO in the culture medium never exceeded 0.01% (v/v), and the same concentration was present in control experiments.

### Cell-proliferation assay

Cell proliferation/viability were determined using the 1-(4,5-dimethylthiazol-2-yl)-3,5-diphenylformazan (MTT) assay [19]. Briefly, the cells were seeded in a 96-well plate at 10,000 cells per well. A day after seeding, the culture medium was changed, and freshly prepared GA was incubated at different doses (0–2,500 nM) for 24 h. Then incubated with MTT for 3 h. Absorbance at 570 nm as a reference wavelength of 630 nm was measured with a spectrophotometric microplate reader (Biotek Synergy H1 Multi Mode Microplate Reader, Winooski, VT, USA). To measure the viable cells, the absorbance values are compared with the control group and calculated as the percentage of viable cells. Viability analyses were carried out in GA-incubated cells to determine the IC_50_ (half-value of the concentration that could lead to a complete inhibition) for the cells. In vitro cultured cells were incubated with GA up to 24 h [20]. IC_50_ values were calculated using the dose-inhibition multiple slope equation with GraphPad Prism 5 program.

### Cell-morphology analysis

Toluidine blue (TB) is a metachromatic and acidophilic dye that can stain acidic tissue components such as carboxylates, sulfates, and phosphate radicals. The production of proteoglycans, one of the extracellular matrix elements, is one of the important indicators of cell viability to show the integrity and morphology of the cell. TB staining can be used to determine the viability of cells [19, 21]. We used it to show the cell viability and cell integrity of the TB staining protocol after incubations. Briefly, the cells were seeded in 24-wells plate at 25 × 10^4^ in a 6-well plate. The following day, the cell culture medium was aspired, and GA was added at different doses (0–2,500 nM) for 24 h. Washed the cells twice with 2 mL phosphate buffered saline (PBS) and fixed them with paraformaldehyde (4%) at least for 30 min. After fixation, it was washed twice and then 0.1% TB solution was added for 30 min. The plates were washed with distilled water for 5 min. Washing was repeated thrice until the TB was cleared. The cells were imaged at 10× magnification using the Zeiss Axio Vert.A1 inverted microscope (Zeiss, Oberkochen, Germany).

### Quantitative real-time PCR experiments

Total mRNA isolation was performed using the commercially available Qiagen kit from the media of MDA-MB-231 and BT-20 cells cultured for 24 h in the presence or absence of GA. The quality and amount of total RNA samples obtained from cell media were measured spectrophotometrically with the Take 3 plate. It was stored at −80 °C until the time it was used. The complementary DNA (cDNA) synthesis reactions were performed using the miScript II RT Kit (Cat. no.: 218161, Qiagen). Expression levels of E-cadherin, N-cadherin, Bax, neuroserpin, and actin beta (ACTB) were determined with the QuantiTect SYBR Green PCR Kit (Qiagen) using the quantitative real-time polymerase chain reaction (qRT-PCR) method by Roche LightCycler 96 instrument (GmBH, Germany). Results were analyzed using the 2^−ΔΔCq^ method. Primers for E-Cadherin, N-Cadherin, Bax, neuroserpin, and ACTB mRNA as normalizers (Qiagen) were designed as following:

**E-cadherin**, 5 complementary DNA-TCCATTTCTTGGTCTACGCC-3ʹ (forward), 5ʹ-CACCTTCAGCCAACCTGTTT-3ʹ (reverse);
**N-cadherin**, 5ʹ-CGCCATCATCGCTATCCTTCTGTG-3ʹ (forward), 5ʹ-AGCCGCTGCCCTCGTAGTCAAA-3ʹ (reverse);
**Neuroserpin**, 5ʹ-TTGTCGACCATCCATTTTTCT-3ʹ (forward), 5ʹ-GCATGACTCGTCCCATGAAT-3ʹ (reverse);
**Bax**, 5ʹ-GAGAGGTCTTTTTCCGAGTGG-3ʹ (forward), 5ʹ-CCTTGAGCACCAGTTTGCTG-3ʹ (reverse);
**ACTB**, 5ʹ-CCACTGGCATCGTGATGG-3ʹ (forward), 5ʹ-GCGGATGTCCACGTCACACT-3ʹ (reverse).

### Determination of intracellular ROS

To determine the oxidative stress induced by GA in the MDA-MB-231 and BT-20 cells, the mean value of intracellular ROS was measured using cells loaded with the redox-sensitive dye 2ʹ,7ʹ-dichlorofluorescin diacetate (DCFH-DA) (Sigma, Schnelldorf, Germany). To measure the intracellular ROS level of the cells, 25 × 10^4^, both cells were placed in each well of the 6-well plate and incubated with GA doses of all groups for 12 h. The cells were washed twice in 2 mL PBS, stained with 50 mM 0.5 μL DCFH-DA in the dark for 30 min, and harvested at 37 °C. The cells were lysed with 1% Triton X-100, and fluorescence was measured using the fluorescence microplate reader at Excitation 485 nm and Emission 530 nm.

To measure the total protein levels, we used duplicate cultures that were subjected to the same treatments. Protein concentrations were detected by a modified Bradford assay; it was measured at 595 nm using Coomassie Plus reagent with bovine serum albumin as a standard. The ROS levels were normalized as arbitrary unit per mg protein and presented as the percentage of control.

### Cell apoptosis DAPI detection

The 4,6-diamidino-2-phenylindole (DAPI) is the most-used fluorescent dye because it binds specifically to DNA. DAPI can be used in a fast and effective analysis to observe DNA fragmentation, a process of apoptosis. Also, cells that do not undergo apoptosis have round nuclei and a single structure, with prominent edges of the nucleus. As a result of the stimulation of apoptosis by GA, the permeability of DAPI increases in the cell membrane and the intensity of bright blue fluorescence enhances due to the condensation of chromosomes in the nucleus [22, 23]. Therefore, DAPI staining can also be used to detect apoptotic cells. Briefly, before the GA incubation, cells were seeded at 25 × 10^4^ in a 6-well plate overnight. After the cells were incubated with the determined doses of GA for 12 h in the 6-well plate, the media was discarded, and the cells were washed in PBS. DAPI stain solution was added to a final concentration of 300 nM and incubated at 37 °C in the dark for 1–5 min, protected from light. We removed the stain solution and washed the cells 2–3 times in PBS. Apoptotic cells were visualized at 20× magnification under a fluorescence microscope using the Zeiss Axio Vert.A1 inverted microscope (Zeiss, Oberkochen, Germany). This measurement was made at least six times in all groups.

### Statistical analysis

Statistical analysis of the results was performed using the GraphPad Prism 5.03 program. Tests were performed in six samples of each group. The results were normalized to the control group. The values were presented as average ± standard deviation (average ± SD). We used the nonparametric Kruskal–Wallis test to determine significant differences between dose groups in each cell line. When a significant difference was found in this test, Dunn’s test was used to evaluate the comparisons between the two groups. For all comparisons, differences were considered statistically significant at *P* < 0.05. All experiments were performed three times, each consisting of duplicate cultures.

## Results

**Figure 1.**
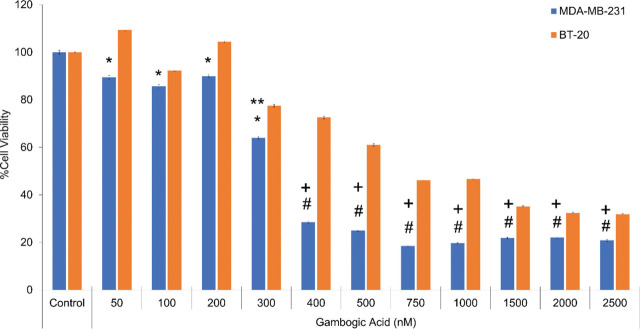
Cytotoxic effect of GA on the BT-20 and MDA-MB-231 cells groups incubated for 24 h (n = 6 in each experiment). Values were mean ± SD. ^*^Values significantly different from control, 50 nM, 100 nM, 200 nM, and 300 nM GA groups in the BT-20 cells (*P* < 0.01). ^**^Values significantly different from control, 50 nM, 100 nM, and 200 nM GA groups in the MDA-MB-231 cells (*P* < 0.001). ^#^Values significantly different from control, 50 nM, 100 nM, 200 nM, and 300 nM GA groups in the MDA-MB-231 and BT-20 cells (*P* < 0.001). ^+^Values significantly different from control, 400 nM, 500 nM, 750 nM, 1,000 nM, 1,500 nM, 2,000 nM, and 2,500 nM GA groups in the BT-20 cells (*P* < 0.001). GA, gambogic acid,

**Figure 2.**
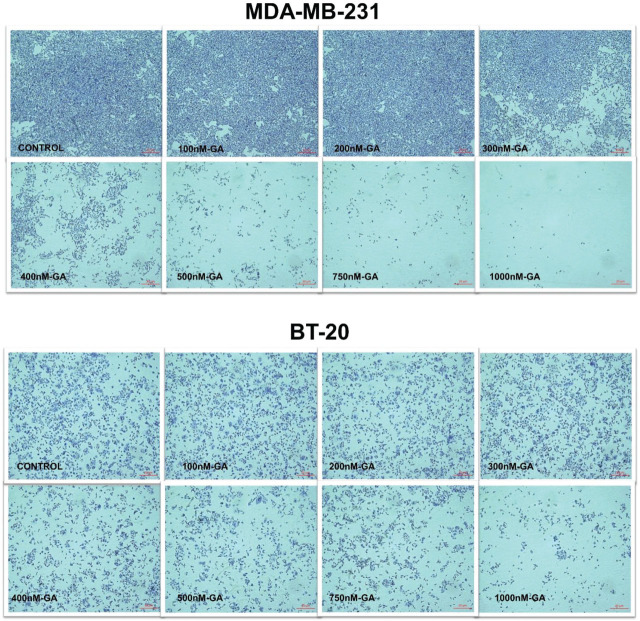
Cell pictures show the cell morphology and viability of MDA-MB-231 and BT-20 control and GA-treated cells (10× magnification). GA, gambogic acid.

Dose-dependent cytotoxicity effects of various concentrations of GA were determined in BT-20 and MDA-MB-231 cells by the MTT assay (**Figure 1**). The IC_50_ values of GA were 315.8 nM and 441.8 nM in the MDA-MB-231 and BT-20 cells for 24 h, respectively. The IC_50_ dose of GA was higher in BT-20 cells than in the MDA-MB-231 cells at 24 h. As shown in **Figure 1**, the viability of MDA-MB-231 cells was diminished by 70% at the IC_50_ dose of the BT-20 cell (*P* < 0.001). It was also found that BT-20 cells were more resistant to GA than the MDA-MB-231 cells (**Figure 1**). Similar to MTT results, TB staining results showed that BT-20 cells and MDA-MB-231 cells decreased in their viability depending on the dose of GA, and that MDA-MB-231 cells were more sensitive to GA treatment compared to BT-20 cells (**Figure 2**).

**Figure 3.**
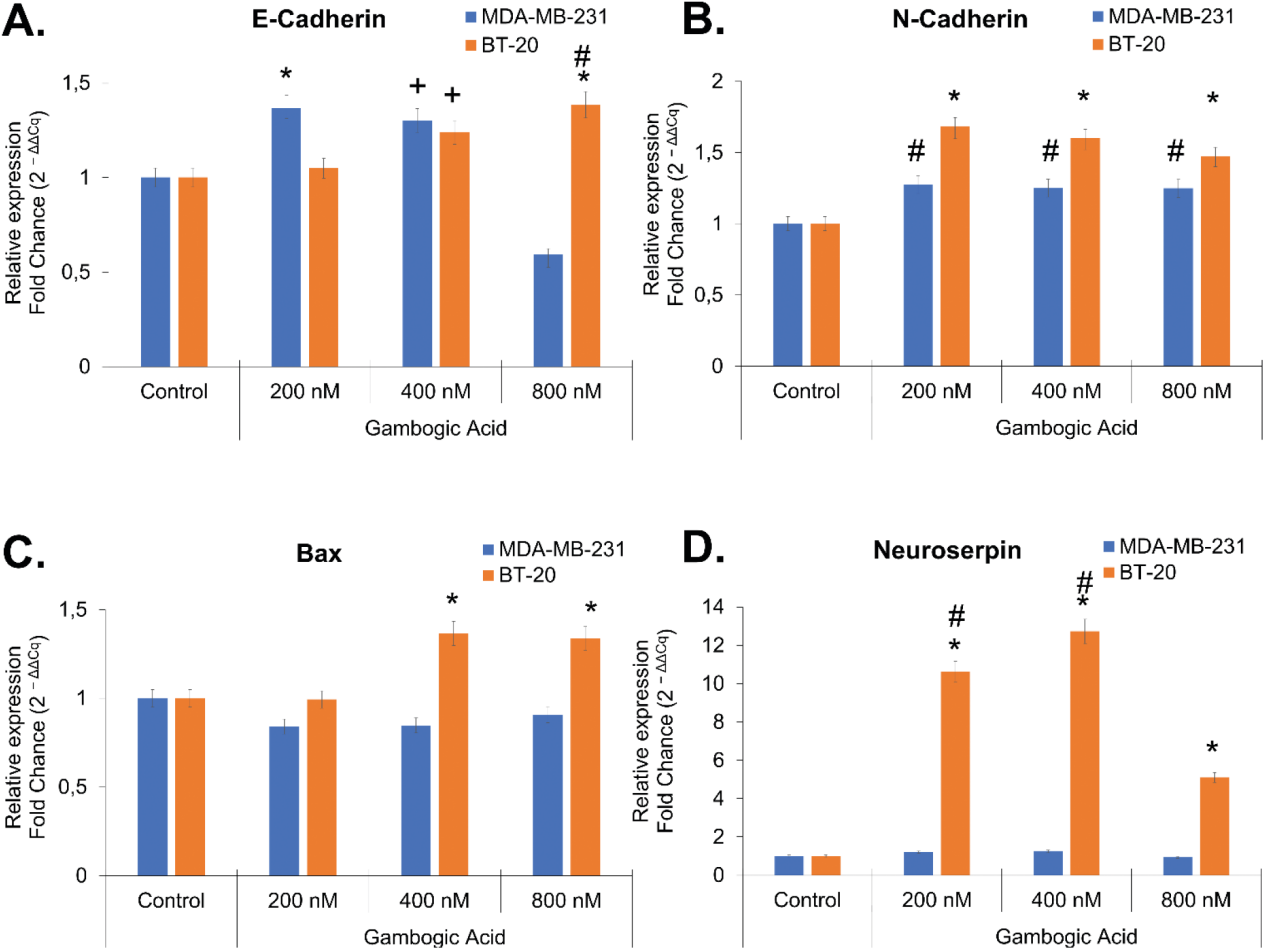
mRNA levels of E-cadherin, N-cadherin, Bax, and neuroserpin in a dose-dependent manner following treatment by qRT-PCR. Data were presented as the mean ± standard deviation (n = 6). **(A)** E-cadherin relative mRNA expression level. ^*^Values significantly different from control and 800 nM GA groups in the MDA-MB-231 cells (*P* < 0.01). ^#^Values significantly different from control, 200 nM, and 400 nM GA groups in the BT-20 cells (*P* < 0.001). ^+^Values significantly different from control group in the MDA-MB-231 and from control and 200 nM GA groups BT-20 cells (*P* < 0.01). **(B)** N-cadherin relative mRNA expression level. ^*^Values significantly different from all groups in the BT-20 and MDA-MB-231 cells (*P* < 0.001). ^#^Values significantly different from control group in the BT-20 and MDA-MB-231 cells (*P* < 0.01). **(C)** Bax relative mRNA expression level. ^*^Values significantly different from control and 200 nM GA groups in the BT-20, and different from all groups in MDA-MB-231 cells (*P* < 0.01). **(D)** Neuroserpin relative mRNA expression level. ^*^Values significantly different from control group in the BT-20, and different from all groups in MDA-MB-231 cells (*P* < 0.001). ^#^Values significantly different from 800 nM GA group in the BT-20 cells (*P* < 0.001). GA, gambogic acid.

It is well known that the loss of E-cadherin is a crucial event for the epithelial–mesenchymal transition (EMT) induction. Also, it protects cancer cells from induced apoptosis [24]. However, any change in E-cadherin expression of GA-induced MDA-MB-231 cells was observed at the 200 nM and 400 nM doses except for the 800 nM dose. Otherwise, MDA-MB-231 cells initially developed a resistance mechanism against GA. This may have decreased the E-cadherin levels to save the cell from apoptosis with a dose of 800 nM (*P* < 0.001) (**Figure 3A**). Therefore, we demonstrated that a 400 nM dose of GA would induce apoptosis for 12 h and the majority of MDA-MB-231 cells died for 24 h (**Figures 3, 5, and 6**). Furthermore, there was no decrease in E-cadherin levels at any dose of GA in BT-20 cells (**Figure 3A**). Upregulation of N-cadherin, another EMT marker, protects cancer cells from apoptosis [25]. There was no increase in N-cadherin levels with any dose of GA in MDA-MB-231 cells (**Figure 3B**). These cells were already more sensitive to even low doses of GA. However, it was observed an approximately 1.5-fold increase in N-cadherin expression levels at all doses of GA in BT-20 cells (*P* < 0.001) (**Figure 3B**). Therefore, we thought that BT-20 cells might be more resistant to GA.

Bax is a main pro-apoptotic member of the Bcl-2 family of proteins as a mitochondrial dysfunction marker in all cells [20, 26]. As presented in **Figure 3C**, compared with the control group, GA stimulation (400 nM and 800 nM) for 24 h significantly increased Bax expression in a dose-dependent manner in BT-20 cells (*P* < 0.001). Moreover, the IC_50_ value of GA was 441.8 nM in BT-20 cells for 24 h. Conversely, Bax expression was not significantly changed in the MDA-MB-231 cells after the treatment of GA.

Neuroserpin, a serine protease inhibitor, is also known as a tissue-specific tumor suppressor gene in the brain. [27]. But, neuroserpin may also be linked to adverse conditions such as the growth, progression, and aggression of many cancers [28, 29]. All doses of GA activated the cell’s survival mechanism in BT-20 cells through neuroserpin increased by approximately 10–12-fold. However, no change in the expression level of neuroserpin was observed at all doses of GA in MDA-MB-231 cells (*P* < 0.001) (**Figure 3D and Figure 7**).

**Figure 4.**
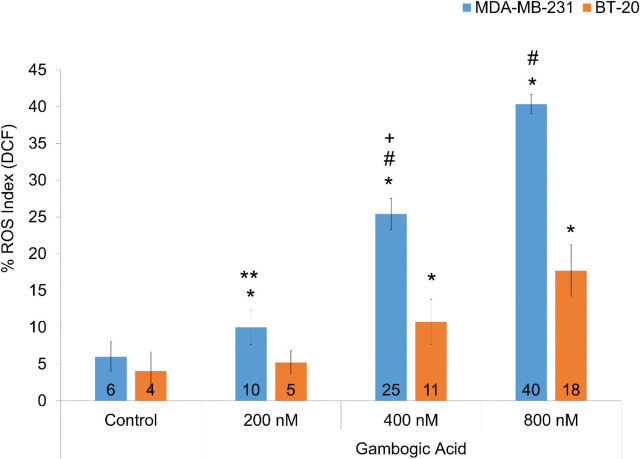
Percentage of ROS index in the MDA-MB-231 and BT-20 cell groups incubated for 12 h (n = 6 in each experiment). Values are mean ± SD. ^*^Values significantly different from control groups in the BT-20 and MDA-MB-231 cells (*P* < 0.01). ^**^Values significantly different from the 200 nM GA group in the BT-20 cells (*P* < 0.01). ^#^Values significantly different from the 400 nM and 800 nM GA groups in the BT-20 cells (*P* < 0.001). ^+^Values significantly different from 200 nM and 800 nM GA groups in the BT-20 and MDA-MB-231 cells (*P* < 0.001). GA, gambogic acid; ROS, reactive oxygen species.

After incubation with all of the GA doses for 12 h, the amount of ROS was measured in both cell lines. ROS generation aggravated in all GA groups in MDA-MB-231 cells. It was shown that ROS generation in GA groups was higher in the MDA-MB-231 than in the BT-20 cells (*P* < 0.001) (**Figure 4**). GA increased ROS generation indirectly. However, neuroserpin levels were dramatically increased in the BT-20 cells, indicating that neuroserpin contributes to reverse the redox imbalance induced by GA and decreased the amount of ROS. Therefore, we thought that the BT-20 cells may be more resistant to GA. Our results indicate that GA disrupted the redox balance, and the apoptotic pathways were activated with GA in TNBC cells.

**Figure 5.**
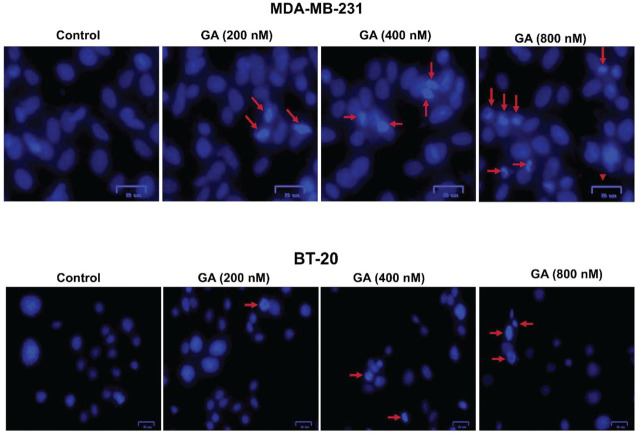
DAPI staining of cells incubated for 12 hours in a dose-dependent manner of GA. DAPI fluorescence images of apoptotic cells with arrows indicating chromatin condensation and high blue fluorescence in the cell nucleus; Magnification ×20; h, hours. GA, gambogic acid.

**Figure 6.**
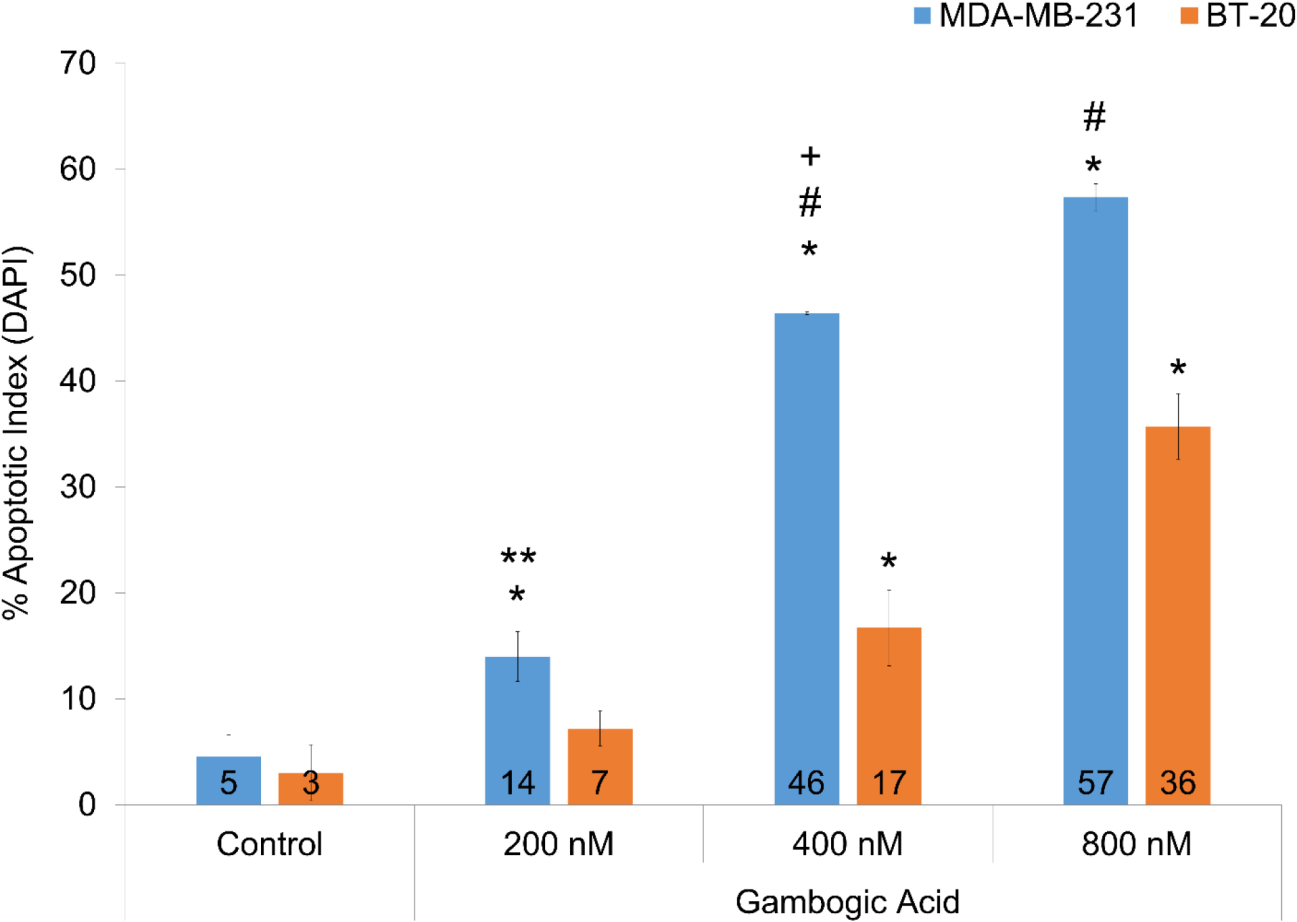
Percentage of apoptotic index in the cell groups incubated for 12 h (n = 6 in each experiment). Shiny DAPI-positive cells were quantified in the various experimental groups. Cells present in 6 high-powered fields (HPF; Magnification ×20) were counted in each experiment and the ratio of apoptotic cells was calculated as the percentage of the whole cell number. Values were mean ± SD. Statistical analysis was performed by one-way analysis of variance with all pairwise multiple comparison procedures done by Dunn’s test. ^*^Values significantly different from control groups in the BT-20 and MDA-MB-231 cells (*P* < 0.01). ^**^Values significantly different from the 200 nM GA group in the BT-20 cells (*P* < 0.01). ^#^Values significantly different from the 400 nM and 800 nM GA groups in the BT-20 cells (*P* < 0.001). ^+^Values significantly different from the 200 nM and 800 nM GA groups in the BT-20 and MDA-MB-231 cells (*P* < 0.001). GA, gambogic acid.

DAPI staining clearly showed induction of apoptosis in MDA-MB-231 and BT-20 cells after GA treatment (**Figure 5**). The cells incubated with GA exhibited clear apoptotic markers that became more pronounced with increased treatment time. We found that apoptosis in GA groups was higher in MDA-MB-231 than BT-20 cells (*P* < 0.001) (**Figure 6**). In the DAPI staining, there was an accumulation in the number of small and agglomerated cells in the nucleus of the 12 h treated cells, indicating that the number of apoptotic cells also heightened due to the increase in the dose of GA.

## Discussion

Chemotherapeutic drugs are vital in the treatment and monitoring of both early-stage and advanced BC. Chemotherapeutic drugs are distributed nonspecifically to all bio compartments within the body. Thus, they systemically damage and kill cancer cells as well as normal cells of the body, causing negative side effects in the short and long terms of therapy. The effects of even the best-developed chemotherapeutic agents cannot be separated on normal and cancerous cells. Otherwise, natural compounds have a very important function as they can be effective on cancer cells without damaging normal cells, and they can overcome many problems such as all side effects of chemotherapeutics and drug resistance. However, researchers have still struggled to unravel the mystery behind the effect of natural compounds on intracellular pathways. The expression of the estrogen receptor, which has triggered effects in cancer cell growth, is excessive in the majority of BC patients.

Wang et al. [30] demonstrated that GA sensitizes cells to doxorubicin, one of the most widely used anticancer drugs as a topoisomerase II inhibitor in ER-positive BC cells. This event was achieved by suppressing P-glycoprotein, an important drug-resistance mechanism and decreasing antiapoptotic surviving expression. However, the underlying mechanisms of GA-induced anti-tumor effects on TNBC cells have not been elucidated. In addition, the molecular drug-resistance mechanism of GA was previously not studied in MBA-MD-231 and BT-20 BC cells that lack HER2 amplification, ER, and PGR expression. In this study, we aimed to search the effects of GA on cell viability and apoptotic pathways in TNBC. According to MTT viability and cytotoxicity results, we determined that BT-20 cells were more resistant to the cytotoxic and apoptotic effects of GA than the MDA-MB-231 cells. The TB photographs and MTT results were compatible with the doses of GA in both cells (**Figures 1 and 2**). The data of our study confirmed the strong cytotoxic effect of GA on the MDA-MB231 cells, but less in the BT-20 cells. It is known that the BT-20 cells are also more aggressive than the MDA-MB-231 cells. Although BT-20 is more resistant, we think that GA for both cell lines can increase the effectiveness of a chemotherapeutic agent. Thus, GA still has the potential to be used as a potential anticancer agent in TNBC at slightly higher doses than ER-positive BC cells.

E-cadherin expression is recognized to play a crucial role in the suppression of breast tumors such as head and neck squamous cell carcinoma aggressive cancers. The loss of E-cadherin may cause tumors to become more aggressive and increase their invasion ability and metastasis [31]. However, Nieman et al. [32] showed that N-cadherin contributes motility of human BC cells regardless of their expression of E-cadherin. According to our results, GA increased the expression of N-cadherin in BT-20 cells. This indicated that BT-20 tended to increase motility and invasion due to the GA effect. In recent studies, it has been shown that GA increases the mRNA of E-cadherin but suppresses the mRNA of N-cadherin in melanoma and lung cancer cells [33, 34]. However, we found that only N-cadherin is slightly upregulated in the GA-induced BT-20 cells, which are more resistant to GA than the MDA-MB-231 cells (**Figures 3A and B**). Thus, we thought that cells developed a different resistance mechanism apart from EMT induction to escape from GA-induced apoptosis.

Bax is found in the cytosol of all mammalian cells, including normal and cancer cells. It has an important function of initiating apoptosis by causing mitochondrial dysfunction and is one of the proapoptotic proteins of the Bcl-2 family that control apoptosis. Any event that triggers Bax increases mitochondrial membrane permeability and leads to cancer cell death by the release of cytochrome *c*, the inevitable factor of apoptosis [20, 35]. In a recent study with Her-2 positive MDA-MB-453 BC cells, researchers also found that GA incubation had no change in Bax levels compared to control [36]. Similarly, we found no significant change in Bax levels in GA-induced apoptosis in MDA-MB-231 cells compared to control, whereas in BT-20 cells, there was a 1.5-fold increase with 400 nM GA compared to control. It has been reported that GA strongly induces apoptosis via an oxidative stress-mediated caspase-3 activation in hepatocellular carcinoma [37]. Also, neuroserpin was shown to attenuate H_2_O_2_-induced oxidative stress in hippocampal neuron cells [38]. Otherwise, neuroserpin is involved in the apoptosis process with the FAS signaling pathway and regulates cell death independently of Bax protein via caspase 3–8 [29, 39]. In light of these facts, it is logical to think that neuroserpin could protect TNBC cells from GA-induced oxidative stress and subsequently apoptosis, independently Bax. Moreover, tumor cells behave as a tight regulation of redox homeostasis to manage higher ROS levels and a low degree of oxidative stress [40]. In a study conducted in prostate cancers, it was shown that GA increased ROS levels further, disrupted cellular redox balance, and induced ferroptosis, a type of apoptosis characterized by the accumulation of iron and lipid peroxides [15]. In our study, we observed that ROS increased in MDA-MB-231 cells, but the amount of ROS decreased with the increase of Bax and neuroserpin expression levels in BT-20 cells.

Neuroserpin has been shown to be associated with cancer that functions as a tissue-specific tumor-suppressor gene in the brain [27]. Recent studies indicate that neuroserpin overexpression is connected with protective outcomes in ischemic stroke, and also neuroserpin administration suppresses the apoptotic pathway in glaucoma [41, 42]. It has been reported that neuroserpin gene expression increases in advanced prostate cancer and hepatocellular carcinoma and worsens the prognosis of the disease [28, 43]. On the other hand, while GA incubation upregulated neuroserpin in BT-20 cells, we did not observe any changes in MDA-MB-231 cells. Therefore, neuroserpin may be associated with the development, progression, and aggressiveness of BT-20 cells.

The differential effect of GA on the BT-20 and MDA-MB-231 cell lines could be due to the epithelial character of these cells. It is well known that neuroserpin functions as a significant protective molecule for metastatic cancer cells from a defense mechanism called blood-brain-barrier (BBB) in the colonization process of brain metastasis [29]. During colonization, metastatic cancer cells with mesenchymal character tend to increase their decreased E-cadherin expression [24]. Therefore, we hypothesized that the expression levels of E-cadherin and neuroserpin could be upregulated by dependent or independent mechanisms in metastatic colonization. Consequently, the BT-20 cells with elevated E-cadherin levels compared to the MDA-MB-231 cells may have higher potential for their ability to acquire resistance to apoptosis-inducing anti-cancer molecules such as GA by increasing neuroserpin.

**Figure 7.**
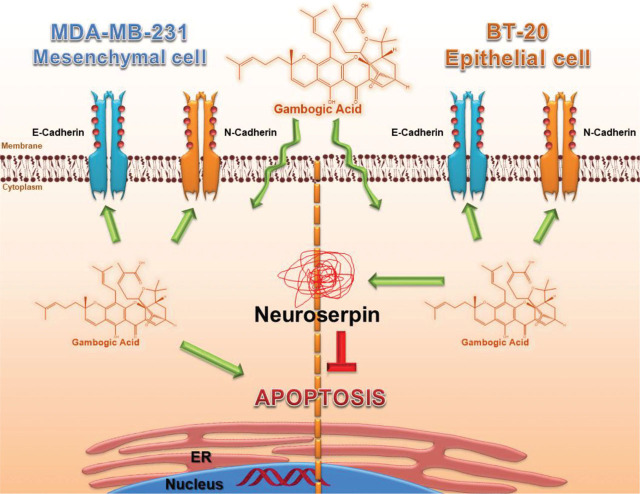
Schematic summary of the changes in the expression profiles of GA-induced apoptotic pathway in the TNBC. GA, gambogic acid; TNBC, triple-negative breast cancer.

There are many studies in literature investigating the effects of natural compounds on ROS-induced apoptosis in cancer. However, there is an increasing debate about whether natural compounds can be used during chemotherapy in patients receiving cancer treatment recently. While natural compounds increase the effects of chemotherapeutic drugs, they could also adversely effects and reduce their bioavailability. In order to shed light on this discussion, we investigated the effects of GA on treatment-resistant BC cells. The findings of the present study indicate that GA induces apoptosis via oxidative stress mechanism in TNBC cells. Also, as neuroserpin acts as a protective factor for cells from oxidative stress [38]. Our results show that there are fewer apoptotic cells and less oxidative stress formation in BT-20 cells. Additionally, GA leads to a dramatic upregulation of Neuroserpin in BT-20 cells compared to MDA-MB-231 cells; no increase in its expression was observed here. We think that the reason for this is the increase in neuroserpin. All these findings clearly indicate that Neuroserpin might be an alleviative effect on GA-induced oxidative stress and subsequent apoptosis in TNBC cells **(Figure 7)**. Accordingly, we hypothesized that neuroserpin may play a role in the resistance of TNBC patients to apoptosis through a mechanism similar to many of the obstacles encountered in the clinic, such as the development of drug resistance. In conclusion, all findings suggest that neuroserpin could be used as a target to alleviate the chemotherapy resistance in TNBC patients.
